# Practical Notes

**Published:** 1890-01

**Authors:** 


### Practical Notes.

### DISINFECTION IN CONTAGIOUS DISEASES AND EPIDEMICS.

The Council of Public Hygiene an1 Health of the Rhone has issued the following instructions relative to disinfection:

1. PRECAUTIONS TO BE TAKEN BY THE ATTENDANTS UPON THE SICK.—Persons caring for the sick shall be provided with garments which must be changed, 1st, when leaving the house; 2nd, during meals; 3rd, when the attendants are obliged to come in contact with healthy persons in the house.

They must wash their hands frequently, especially before meals, with soap and large quantities of water, renewed several times.

2. DISINFECTION OF UTENSILS USED BY THE SICK.—Cups, bowls, plates, spoons, etc., used by the sick, must be carefully cleaned with boiling water and they must not be used by other persons.

The remains of food left by the sick must not be eaten by healthy persons.

3. DISINFECTION OF EXCREMENTITIOUS MATTERS, ETC.—Vomited matters and the dejecta must be immediately mixed with a glassful of an acidulated solution of sulphate of copper, (water 100 grm., sulphate of copper 2 grm., ordinary sulphuric acid 4 grm.) and then thrown into the water closet. The vessels which are used to receive these matters must be first rinsed and then immediately treated with, first, another glassful of the acidulated solution of sulphate of copper and then with a large quantity of ordinary water.

In the country these matters must be buried, after disinfection, far from all water supplies for domestic uses.

If matters have been spread upon the floor they must be absorbed by sawdust, which must be immediately burned. The remainder must then be removed by a sponge or clout impregnated with carbolic acid, to be disposed of as hereinafter described.

If bedding, napkins or handkerchiefs have been soiled, these articles must be immersed in a solution of carbolic acid, and treated as will presently be described.

4. DISINFECTION OF LINEN DURING THE COURSE OF THE DISEASE.—Body linen, napkins, handkerchiefs and bedding must be disinfected in the house before being sent to the laundry. For this purpose whether soiled or not, when changed they must first be immersed for half a day at least in a tub filled with a two per cent. carbolic solution, they must then be lightly wrung out and placed in boiling water for twenty minutes or half an hour, they must then be put in the wash or sent to the laundry.

It is very important to remember that all discharges from the patient must not be permitted to dry upon the linen.

Treatment by the disinfecting oven is preferable to boiling when this is possible.

The carbolic acid solution thus employed must be turned into the water closet, and not into the sinks.

Linen and dressings of little value must be burned; otherwise they must be disinfected in the same manner as body linen.

5. DISINFECTING OF CLOTHING, BEDDING, CARPETS AND HANGINGS AT THE END OF THE DISEASE.—The clothing of the sick, the bedding (coverings, mattress, pillows), and the carpet and curtains of the sick room must be enveloped in cloths moistened with water or a carbolized solution and then be conveyed to a disinfecting oven where they are subjected for twenty minutes to the action of steam without pressure.

These articles must not be returned to their places until the room has been disinfected.

Wearing apparel must also be treated in this manner, even if it is to be given away or sold.

6. DISINFECTION OF THE FURNITURE FOR THE SICK ROOM.—This procedure includes several steps which must always be taken in the following order:

1. Moisten the floor uniformly with water.

2. Carefully wipe the ceiling, the walls or the hangings with a cloth slightly dampened so as to remove the dust.

3. Furniture of small value, such as common chairs, etc., must be liberally treated with a sublimate solution (1-1000).

The inside of night chairs should always be so treated.

4. In the more important furniture, such as beds and hair mattresses, all the joints and crevices must be treated with the solution of sublimate as in the destruction of vermin; waxed and varnished surfaces must be wiped with a cloth impregnated with oil.

5. Upholstered chairs must be beaten and then rubbed with a brush dipped in the sublimate solution.

6. The floor of the chamber and the wainscoting and walls up to a height of 6 feet must be washed and cleaned with brushes and sponges dipped in the sublimate solution.

7. Not later than two hours afterward a second liberal washing must be given with sponges dipped this time in an alkaline solution (water 100 grm., carbonate or crystals of commercial soda 10 grm).

At the same time the less valuable furniture which has previously been treated with the sublimate solution, must now be well washed with the alkaline solution.

8. The excess of the solution must be mopped up, the room well ventilated so as to dry the walls and floor as quickly as possible.

The preceding measures ought to insure sufficient security.

In case, however, the rooms to be disinfected can be dispensed with for 48 hours it is well to complete the disinfection by fumigation with sulphurous acid.

For this purpose, before drying the room, an iron vessel is placed in the middle of the floor; into this is placed flour of sulphur in the proportion of 15 to 20 grm. per cubic metre of the space to be disinfected. To avoid danger of fire the vessel containing the sulphur should be placed in a large receptacle containing 5 or 6 centimetres of water. The chimney is then stopped and strips of paper are pasted over all cracks where the vapor might escape. Metallic objects which can not be removed are covered with grease. Alcohol is then poured upon the sulphur and ignited. The door of the apartment is then closed and the crevices covered with strips of paper. Sulphurous acid gas is evolved and should be confined for 12 hours at least. The next day the room is well aired. It may be reoccupied as soon as the gas which generally provokes irritation of the eyes and throat is no longer noticed—this will generally be in about 24 hours after the windows have been opened.

Finally, when possible, it will be advisable to re-paper or re-paint the walls as the case may be.—*La Prov. Med.*

